# Heat Shock Protein 27 Affects Myeloid Cell Activation and Interaction with Prostate Cancer Cells

**DOI:** 10.3390/biomedicines10092192

**Published:** 2022-09-05

**Authors:** Debora Singer, Verena Ressel, Matthias B. Stope, Sander Bekeschus

**Affiliations:** 1 ZIK *plasmatis*, Leibniz Institute for Plasma Science and Technology (INP), Felix-Hausdorff-Str. 2, 17489 Greifswald, Germany; 2 Clinic and Policlinic for Urology, Greifswald University Medical Center, Ferdinand-Sauerbruch-Str., 17475 Greifswald, Germany; 3 Department of Gynecology and Gynecological Oncology, University Hospital Bonn, 53127 Bonn, Germany

**Keywords:** cytokines, HL-60, Hsp27, monocytes, ROS, THP-1

## Abstract

Heat shock proteins are cytoprotective molecules induced by environmental stresses. The small heat shock protein 27 (Hsp27) is highly expressed under oxidative stress conditions, mediating anti-oxidative effects and blocking apoptosis. Since medical gas plasma treatment subjects cancer cells to a multitude of reactive oxygen species (ROS), inducing apoptosis and immunomodulation, probable effects of Hsp27 should be investigated. To this end, we quantified the extracellular Hsp27 in two prostate cancer cell lines (LNCaP, PC-3) after gas plasma-induced oxidative stress, showing a significantly enhanced release. To investigate immunomodulatory effects, two myeloid cell lines (THP-1 and HL-60) were also exposed to Hsp27. Only negligible effects on viability, intracellular oxidative milieu, and secretion profiles of the myeloid cells were found when cultured alone. Interestingly, prostate cancer-myeloid cell co-cultures showed altered secretion profiles with a significant decrease in vascular endothelial growth factor (VEGF) release. Furthermore, the myeloid surface marker profiles were changed, indicating an enhanced differentiation in co-culture upon Hsp27 treatment. Finally, we investigated morphological changes, proliferation, and interaction with prostate cancer cells, and found significant alterations in the myeloid cells, supporting the tendency to differentiate. Collectively, our results suggest an ambiguous effect of Hsp27 on myeloid cells in the presence of prostate cancer cells which needs to be further investigated.

## 1. Introduction

Cells can be subjected to various environmental stresses, such as heat or chemical stress conditions. In order to protect themselves from damage caused by these stress events, small molecules like heat shock proteins are formed which serve as chaperones for other proteins [1]. A major molecule strongly induced by stress conditions is the small heat shock protein 27 (Hsp27), which is also induced by oxidative stress conditions [2,3,4]. Oxidative stress follows on from increased levels of reactive oxygen species (ROS) [5], which can damage the cell either directly by oxidizing biomolecules [6], or by impairing redox signaling [7], both leading to cell death. To prevent this, Hsp27 mediates anti-apoptotic effects by inhibiting caspase activation [8], promotes the proteasomal degradation of oxidized proteins [9], and can regulate the actin cytoskeleton [10]. Furthermore, Hsp27 can act directly as an antioxidant by raising glutathione levels [11] and decreasing iron levels [12].

Sources of oxidative stress can either be the endogenous production of ROS, as in cellular respiration or normal metabolism, or exogenous as caused by radiation or microbial infection [13]. Oxidative stress can also be intentionally applied to cells, as is the case, for example, in cancer therapy. Chemotherapy can induce oxidative stress in the cells [14]. Unfortunately, this could also impair the effectiveness of the therapy since high levels of Hsp27 are associated with chemotherapy resistance [15]. Another application currently being researched as a cancer therapy option which uses oxidative stress, is the treatment with medical gas plasma [16]. Here, a range of ROS, such as hydrogen peroxide (H_2_O_2_), hydroxyl radical, superoxide, and reactive nitrogen species (RNS) are subjected to the cells [17], leading to oxidative stress [18], apoptosis induction [19], and inflammatory changes [20] as, for example, found in prostate cancer cells [21]. Possible mechanisms involved in the gas plasma-induced cancer cell death are interactions with calcium signaling, endoplasmic reticulum (ER) stress, and altered intracellular signaling due to increased intracellular RONS [22], as well as changes in the tumor’s microenvironment by affecting immune cells directly or indirectly via the induction of pro-inflammatory signals released by the cancer cells [23].

Little is known about a possible induction of Hsp27 in gas plasma-treated cancer cells, especially the Hsp27-related effects on immunomodulation. Therefore, in this study we aimed to investigate the role of Hsp27 on myeloid cells, focusing on their interaction with prostate cancer cells.

## 2. Materials and Methods

### 2.1. Cell Culture

Two prostate cancer cell lines, LNCaP (ATCC: CRL-1740) and PC-3 (ATCC: CRL-1435), as well as two myeloid cell lines, THP-1 (ATCC: TIB-202) and HL-60 (ATCC: CCL-240) were used in this study. Cells were cultured in Roswell Park Memorial Medium (RPMI) 1640 supplemented with 10% fetal bovine serum, 2% L-glutamine, and 1% penicillin/streptomycin under standard conditions (37 °C, 5% CO_2_, 95% humidity).

### 2.2. Oxidant Exposure and Hsp27 Quantification

For Hsp27 induction experiments, 3 × 10^4^ cells (LNCaP) or 1 × 10^4^ cells (PC-3) in 500 µL of fully-supplemented cell culture medium were seeded to 24-well plates. For oxidative stress induction, cells were treated with the atmospheric pressure argon plasma jet kINPen (neoplas med, Greifswald, Germany) for 10 s at a feed gas flux of four standard liters per minute. Untreated cells served as controls. Cells were incubated, and supernatants were collected after 24 h, 48 h, 72 h, or 96 h and stored for subsequent enzyme-linked immunosorbent assays (ELISA). Heat shock protein 27 (Hsp27) concentration was measured using the Human Hsp27 DuoSet ELISA kit (Cat# DY1580; RnD Systems, Wiesbaden, Germany) according to the manufacturer’s protocol.

### 2.3. Metabolic Activity and Viability

The resazurin-based assay was used to determine the cells’ metabolic activity after treatment with 10,000 pg/mL Hsp27 (Cat# ADI-SPP-715-F; Enzo Life Sciences, Lörrach, Germany) or an equivalent volume of cell culture medium only, added to prostate cancer cell and myeloid cell monocultures, as well as co-cultures. A final concentration of 100 µM resazurin was added to the cells and incubated for 4 h. The fluorescence of the resazurin reduction product, resorufin, generated by metabolically active cells, was quantified using the Infinite F200 plate reader (Tecan, Männedorf, Switzerland) at λ_ex_ 535 nm and λ_em_ 590 nm. To determine the viability of monocultured myeloid cells, live-dead discrimination was performed using 4′,6-diamidino-2-phenylindole (DAPI, 1 µM; Cat# 422801; BioLegend, Amsterdam, The Netherlands) staining and analyzed by flow cytometry (CytoFLEX S; Beckman-Coulter, Krefeld, Germany).

### 2.4. Analysis of Oxidation and Mitochondria

To assess intracellular ROS generation, myeloid cells were stained with either chloromethyl 2′,7′-dichlorodihydrofluorescein diacetate (CM-H_2_DCF-DA, 1 µM; Cat# C6827; Thermo Fisher Scientific, Dreieich, Germany) or 3′-(p-aminophenyl) fluorescein (APF, 1 µM; Cat# A36003; Thermo Fisher Scientific). Following staining, 2 × 10^4^ stained cells were seeded into 24-well plates and subsequently treated with Hsp27. To detect the effects on mitochondrial membrane potential, the myeloid cells were stained with mitotracker orange (MTO, 1 µM; Cat# M7511; Thermo Fisher Scientific) before adding Hsp27. DAPI (1 µM) was used in all three assays to distinguish viable from dead cells. The mean fluorescence intensity (MFI) of dichlorofluorescein (DCF) or APF and MTO was analyzed after 4 h or 24 h, respectively, using flow cytometry.

### 2.5. Chemokine, Cytokine, and Growth Factor Quantification

Multiplex chemokine, cytokine, and growth factor quantification in the collected cell culture supernatants were performed 96 h after Hsp27 treatment using a customized bead-based assay (LEGENDplex; Cat# B248224; BioLegend) according to the manufacturer’s protocol. The MFI of the bead populations was measured using flow cytometry. Total analyte concentrations were calculated against a known standard containing arginase, chemokine (C-C motif) ligand 17 (CCL17), chemokine (C-X-C motif) ligand 1 (CXCL1), CXCL10, interleukin 6 (IL-6), IL-8, tumor growth factor-beta (TGF-β), and vascular endothelial growth factor (VEGF), using a 5-log fitting with LEGENDplex software 8.0 (Vigenetech, Carlisle, PA, USA).

### 2.6. Co-Cultures

For co-culture experiments, 96-well plates were coated with 0.01% poly-l-lysine before seeding 2.5 × 10^3^ cells (LNCaP) or 1.25 × 10^3^ cells (PC-3) in 100 µL of fully supplemented cell culture medium. Subsequently, an equal number of THP-1 or HL-60 cells in 20 µL cell culture medium were added to the prostate cancer cells. Following this, 10,000 pg/mL Hsp27 was added to the co-cultures, and cells were incubated for up to 96 h, depending on the downstream assay.

### 2.7. Cell Surface Marker Expression Analysis

Multicolor flow cytometry was used to analyze the expression of several surface markers on the myeloid cells for both monocultures and co-cultures 96 h after Hsp27 treatment. Cells were harvested and transferred to 96-well V-bottom plates, washed and stained with fluorescently-labeled monoclonal antibodies (100 ng/mL) targeting the following surface markers: cluster of differentiation (CD)11b, CD11c, CD14, CD16b, CD32, CD45RA, CD55, CD69, CD71, CD163, human leukocyte antigen (HLA-)ABC, and P2Y purinoceptor 2 (P2Y2) (Table 1). After 15 min of incubation at room temperature in the dark, cells were washed, and DAPI (1 µM) was added to determine live-cell populations. Fluorescence intensities were acquired using a CytoFLEX S flow cytometer.

### 2.8. Live-Cell High-Content Imaging

Prostate cancer cells were stained with CellTrace Violet (1 µM; Cat# C34557; Thermo Fisher Scientific, Dreieich, Germany) prior to the addition of CellTrace Far Red-stained (1 µM; Cat# C34564; Thermo Fisher Scientific) myeloid cells, as described above for the co-culture assays. SYTOX Green (0.1 µM; Cat# S7020; Thermo Fisher Scientific) was added to detect terminally-dead cells. Live-cell imaging was performed using the high-content imaging device, Operetta CLS (PerkinElmer, Hamburg, Germany), preheated to 37 °C and set to 5% CO_2_. Images were acquired 24 h, 48 h, 72 h, and 96 h after Hsp27 addition using a 20× (NA 0.4) air objective (Zeiss, Jena, Germany). Channels were brightfield, digital phase-contrast, CellTrace Violet (λ_ex_ 405 nm, λ_em_ 465 nm), SYTOX Green (λ_ex_ 475 nm, λ_em_ 525 nm), and CellTrace Far Red (λ_ex_ 630 nm, λ_em_ 680 nm).

### 2.9. Statistical and Software Analysis

Statistical analysis was performed using Prism 9.4.1 (Graphpad Software, San Diego, CA, USA). Unpaired two-tailed *t*-test or one-way analysis of variance (ANOVA) followed by Šídák’s multiple comparisons test were used to compare treated against control groups. The level of significance was indicated as follows: *p* < 0.05 (*), *p* < 0.01 (**), and *p* < 0.001 (***). Flow cytometry data were analyzed using Kaluza analysis 2.1.1 (Beckman-Coulter), except for cytokine data which were analyzed with LEGENDplex software 8.0. High-content images were analyzed using Harmony 4.9 (PerkinElmer,) software.

## 3. Results

### 3.1. Hsp27 Is Induced in Prostate Cancer Cells upon Oxidative Stress Exposure

**Figure 1:**
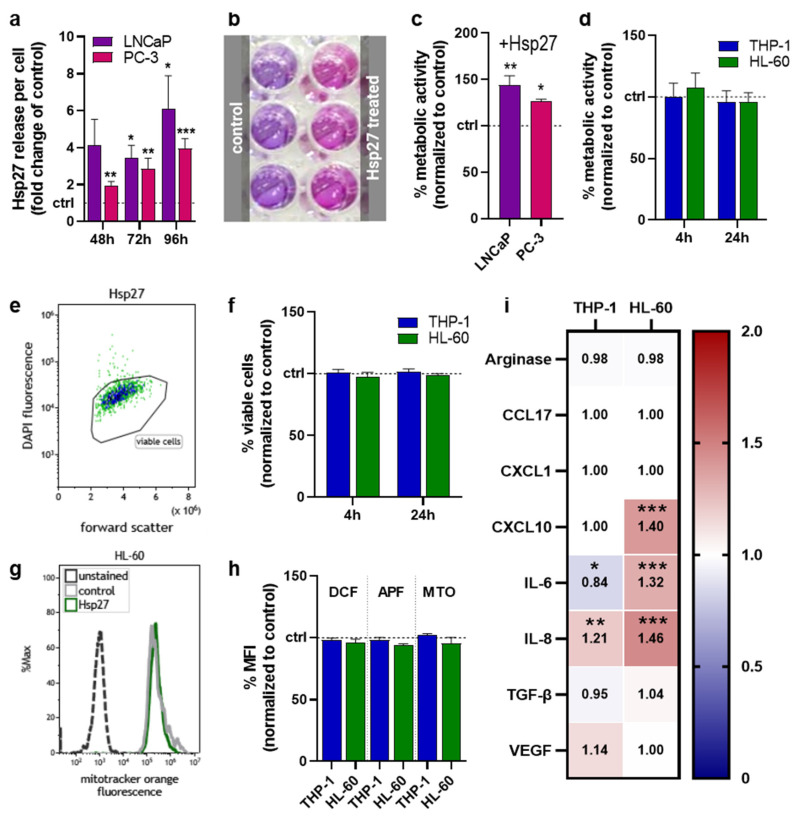
**Oxidative stress-induced Hsp27 and its effects in individual cell lines.** (**a**) fold-change increase in oxidative stress-induced Hsp27 release after 48 h, 72 h, and 96 h in LNCaP and PC-3 cells; (**b**) representative photograph of the resazurin-based metabolic activity assay; (**c**,**d**) normalized metabolic activity of prostate cancer cells 96 h (**c**) and myeloid cells directly (4 h) and 24 h (**d**) following exposure to Hsp27; (**e**) representative flow cytometry dot plot of Hsp27-treated HL-60 cells; (**f**) normalized number of viable myeloid cells determined via flow cytometry directly and 24 h after Hsp27 exposure; (**g**) representative flow cytometry overlay histograms of mitotracker orange fluorescence in HL-60 cells; (**h**) normalized mean fluorescence intensity of dichlorofluorescein (DCF), aminophenyl fluorescein (APF), and mitotracker orange (MTO) in myeloid cells determined by flow cytometry; (**i**) normalized chemokine, cytokine, and growth-factor secretion profile 96 h after Hsp27 exposure in myeloid cells (additional soluble mediators investigated were below the limit of detection, including IL-1β, TNF-α, IFN-γ, IL-12p70, and IL-10). Data are representative of at least three experiments; statistical analysis was performed using one-way (**c**) or two-way ANOVA (**i**) with *p* < 0.05 (*), *p* < 0.01 (**), and *p* < 0.001 (***).

LNCaP and PC-3 cells were subjected to oxidative stress by atmospheric pressure argon plasma jet treatment. The release of heat shock protein 27 (Hsp27) was quantified (Figure 1a), showing a strong increase in both prostate cancer cell lines compared to untreated controls. The extent of extracellular Hsp27 induction also increased with the incubation time after oxidative stress exposure. To analyze the consequences in prostate cancer cells as well as myeloid cells, while excluding effects mediated by the gas plasma treatment per se, cells were treated with Hsp27 alone in the following experiments. To investigate whether Hsp27 would affect the prostate cancer cells’ metabolic activity, the resazurin assay (Figure 1b) was performed. Compared to the untreated controls, Hsp27-treated LNCaP and PC-3 cells showed a significantly increased metabolic activity 96 h after treatment (Figure 1c), suggesting a protective and growth-supportive effect on the cancer cells. To investigate if the released Hsp27 could affect myeloid cells, THP-1 and HL 60 cells were cultured with Hsp27. Metabolic activity was measured shortly after (4 h) and 24 h after treatment, but neither cell line showed any noteworthy changes compared to the controls (Figure 1d). Viability was assessed via flow cytometry using DAPI staining to discriminate viable from dead cells (Figure 1e). The viability of both cell lines was not affected directly (4 h) or 24 h after Hsp27 treatment (Figure 1f). In a following step, the intracellular oxidative milieu of the myeloid cells was investigated using the fluorescent redox-sensitive dyes DCF, APF, and the mitochondrial membrane potential dependent dye MTO, by flow cytometry (Figure 1g). In THP-1 cells, none of the probes showed changes in their MFI upon Hsp27 treatment, whereas in HL-60 cells, a very slight decrease in all three probes was detected (Figure 1h). The immunomodulatory effects of Hsp27 on myeloid cells were investigated by the quantification of several chemokines, cytokines and growth factors in the cell culture supernatants collected 96 h after treatment (Figure 1i). The secretion of arginase, CCL17, CXCL1, and TGF-β was not altered in either cell line. Additional soluble mediators investigated were below the limit of detection, including IL-1β, tumor necrosis factor (TNF-)α, interferon (IFN-)γ, IL-12p70, and IL-10 (data not shown). In THP-1 cells, IL-6 and IL-8 secretion significantly decreased and increased, respectively, with all other soluble mediators remaining unchanged. By contrast, HL-60 cells showed a significant increase in CXCL10, IL-6, and IL-8 secretion, while the other targets were unaffected.

### 3.2. Hsp27 Altered Secretion Profiles of Prostate Cancer Cell–Myeloid Cell Co-Cultures

**Figure 2:**
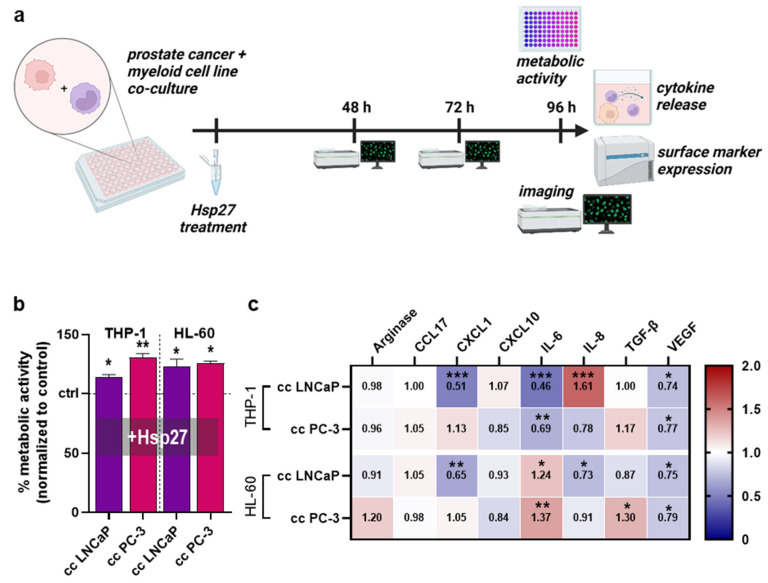
**Co-culture metabolic activity and release profiles.** (**a**) study protocol of the co-culture of prostate cancer cell lines with myeloid cell lines treated with Hsp27; (**b**) normalized metabolic activity of Hsp27 treated co-cultures; (**c**) normalized chemokine, cytokine, and growth-factor secretion profile 96 h after Hsp27 exposure in co-cultures. Data are representative of at least three experiments; statistical analysis was performed using one-way ANOVA (**b**) with *p* < 0.05 (*), *p* < 0.01 (**), and *p* < 0.001 (***).

To investigate whether Hsp27 release alters the interplay between prostate cancer cells and myeloid cells, these cell types were co-cultured and treated with Hsp27, followed by different assays (Figure 2a). After 96 h from Hsp27 treatment, the metabolic activity of all co-cultures had significantly increased compared to untreated controls that had not received Hsp27 (Figure 2b). Here, Hsp27 had the largest effect on the co-culture of PC-3 cells with THP-1 cells, which suggested, in the light of the monoculture results (Figure 1d), accelerated growth of the tumor cells. To understand the secretion profiles which were possibly linked to this effect, the chemokine, cytokine, and growth factor secretions of the co-culture supernatants were measured 96 h after Hsp27 treatment and normalized to the untreated co-culture controls that had not received Hsp27 (Figure 2c). All co-culture combinations had mainly differing secretory profiles, with only VEGF showing a similar and significant decrease in all four co-cultures. CCL17 was barely affected in any condition. The largest effects were found in the co-cultures of THP-1 and LNCaP cells, showing significantly decreased CXCL1 and IL-6 secretion while having a strong IL-8 secretion increase. Co-cultured THP-1 and PC-3 cells showed significantly decreased IL-6 secretion. LNCaP and HL-60 co-cultures showed significantly decreased secretion for CXCL1 and IL-8 and significantly increased IL-6. In PC-3 and HL-60 co-cultures, the Hsp27 treatment significantly increased the secretion of IL-6 and TGF-β. These data provided evidence that Hsp27 modulated the tumor cell–myeloid cell interaction, showing a consistent decrease in VEGF across all conditions, decreased CXCL1 for LNCaP co-cultures, and decreased IL-6 for THP-1 co-cultures, with increased IL-6 for HL-60 co-cultures.

### 3.3. Hsp27 Altered Surface Marker Profiles of Prostate Cancer–Myeloid Cell Co-Cultures

**Figure 3:**
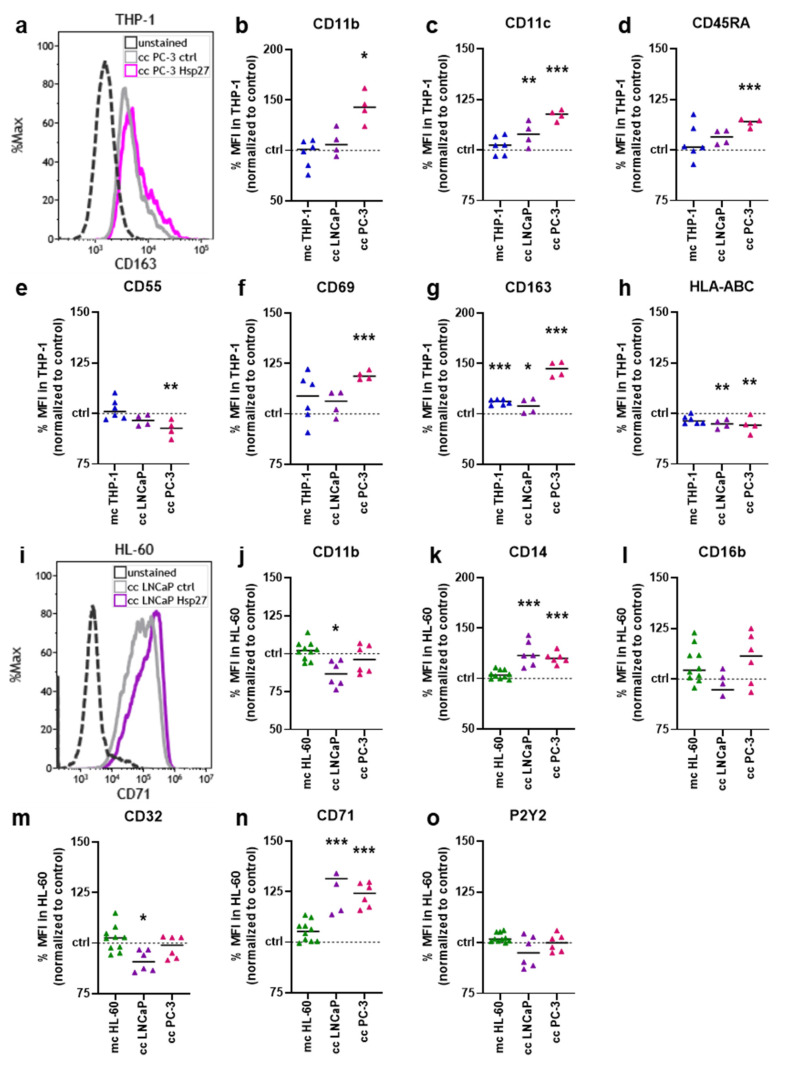
**Hsp27-modified surface marker expression on myeloid–prostate cancer cell co-cultures.** (**a**) representative flow cytometry overlay histograms of CD163 fluorescence in THP-1 cells co-cultured with PC-3 cells; (**b**–**h**) normalized mean fluorescence intensities of THP-1 cells obtained from monoculture (mc) or prostate cancer co-cultures (cc) 96 h after Hsp27 exposure, determined using multicolor flow cytometry; (**i**) representative flow cytometry overlay histograms of CD71 fluorescence in HL-60 cells co-cultured with LNCaP cells; (**j**–**o**) normalized mean fluorescence intensities of HL-60 cells obtained from monoculture (mc) or prostate cancer co-cultures (cc) 96 h after Hsp27 exposure, determined using multicolor flow cytometry. Data are representative of at least three experiments; statistical analysis was performed using one-way ANOVA with *p* < 0.05 (*), *p* < 0.01 (**), and *p* < 0.001 (***).

To investigate whether Hsp27 addition impacted the THP-1 surface expression profile, seven surface markers were analyzed using flow cytometry (Figure 3a). Hsp27-treated THP-1 cells cultured alone and co-cultured together with prostate cancer cells were examined and normalized to the respective untreated controls. The cluster of differentiation (CD)11b was significantly increased in the co-culture with PC-3 cells (Figure 3b). The expression of CD11c was significantly increased in both co-cultures (Figure 3c). CD45RA expression was again only significantly increased in the PC-3 co-culture (Figure 3d). A slight, but significant decrease in CD55 was found in the PC-3 co-culture (Figure 3e). The expression of CD69 was increased in all culture setups, but only significantly in the PC-3 co-culture (Figure 3f). CD163 expression was slightly, but significantly increased in monoculture and LNCaP co-culture, and strongly increased in the PC-3 co-culture (Figure 3g). A slight, but significant decrease of the expression of human leukocyte antigens (HLA-)ABC was observed in all three culture conditions, being significantly decreased in both co-cultures (Figure 3h). Likewise, the expression profile of six different surface markers on HL-60 cells was examined by flow cytometry (Figure 3i). CD11b expression was slightly, but significantly decreased in the HL-60 LNCaP co-culture (Figure 3j). The expression of CD14 was significantly increased in both co-cultures (Figure 3k), while CD16b was not altered in any condition (Figure 3l). The expression of CD32 was modestly, but significantly decreased in the LNCaP co-culture (Figure 3m). A strong and significant increase in CD71 expression was found in both co-cultures (Figure 3n). The P2Y purinoceptor 2 (P2Y2) was not affected significantly in any of the HL-60 culture conditions (Figure 3o). These results indicated that the addition of Hsp27 profoundly changed the surface marker expression profiles of two different myeloid cell lines co-cultured with two different prostate cancer cell lines. In this regard, the co-culture had the strongest effect as did the myeloid cells monoculture (i.e., in the absence of tumor cells), with one modest exception being CD163 in THP-1 cells not showing a significant surface expression following Hsp27 exposure.

### 3.4. Myeloid Cell Proliferation and Interaction with Prostate Cancer Cells Were Mitigated upon Hsp27 Treatment

**Figure 4:**
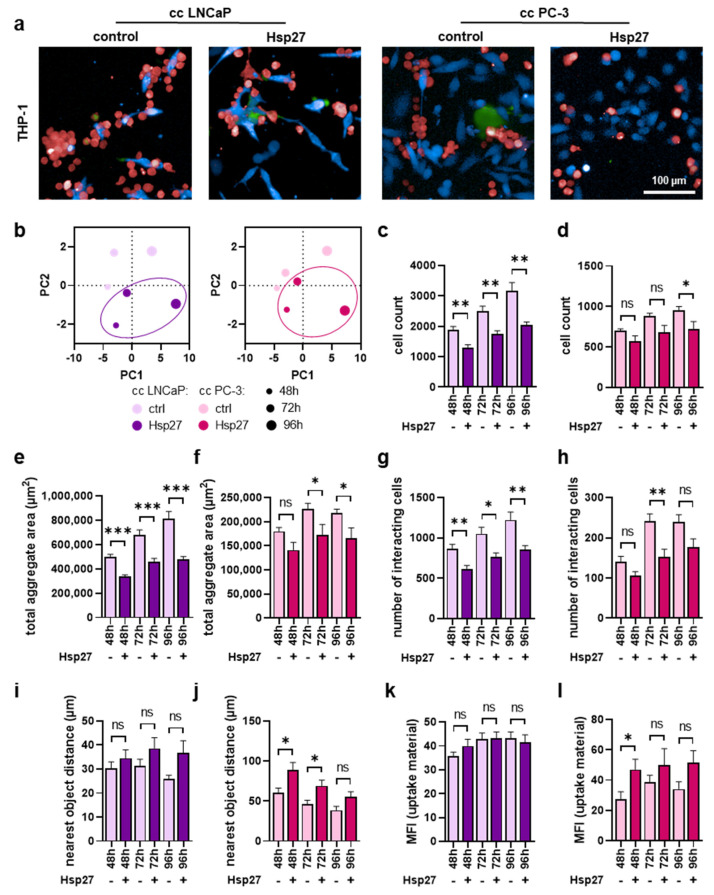
**High-content imaging of THP-1 cells co-cultured with prostate cancer cells under Hsp27 treatment.** (**a**) representative overlay images of untreated (control) and Hsp27-treated co-cultures (cc) of CellTrace Violet-labeled prostate cancer cells (blue) and CellTrace Far Red-labeled THP-1 cells (red), with SYTOX Green (green) as dead cell marker at 96 h; (**b**) PCAs of several morphology properties quantified by algorithm-based image analysis of THP-1 cells; (**c**–**l**) algorithm-based image quantification of THP-1 total cell count (**c**,**d**), the total aggregate area in µm^2^ (**e**,**f**), number of cells interacting with prostate cancer cells (**g**,**h**), distance to the nearest prostate cancer cell in µm (**i**,**j**), and mean fluorescence intensity (MFI) of dead prostate cancer cell material internalized by THP-1 cells (**k**,**l**). Data are presented as mean + SE. Statistical analysis was performed using unpaired, two-tailed t-test with *p* < 0.05 (*), *p* < 0.01 (**), and *p* < 0.001 (***). ns = not significant. Scale bar is 100 µm.

Hsp27 treatment altered myeloid cell secretion and surface marker profiles in co-cultures with prostate cancer cells. Hence, the next question was how would Hsp27 affect the interaction of myeloid cells with prostate cancer cells. Therefore, co-cultured cells were labeled using differential fluorescent staining and analyzed using live-cell imaging up to four days after treatment, followed by the investigation of several parameters using algorithm-based quantitative image analysis. THP-1 cells co-cultured with LNCaP or PC-3 upon Hsp27 treatment (Figure 4a) showed several alterations when compared to the untreated controls. Principal component analysis (PCA) of several morphology properties revealed differences in both co-cultures following a resembling profile (Figure 4b). THP-1 cell counts indicated a significant decrease in the LNCaP co-culture (Figure 4c), as well as in PC-3 co-culture (Figure 4d). The formation of THP-1 aggregates was analyzed and evaluated as the sum area of aggregates found per well within the imaged fields of view. Here, a significant decrease was found in both co-cultures (Figure 4e,f). Similarly, a decrease in the number of THP cells interacting with prostate cancer cells was observed in both co-cultures (Figure 4g,h), as analyzed using algorithms to detect direct cell-cell interactions based on fluorescence signals. This reduced interaction was also visible as an increase in the mean distance of THP-1 cells from neighboring prostate cancer cells (Figure 4i,j). To model phagocytic activities of THP-1 cells, the uptake of dead prostate cancer cell material was quantified, showing no alterations in LNCaP co-cultures (Figure 4k), but a significantly increased uptake of PC-3 material after 48 h (Figure 4l).

**Figure 5:**
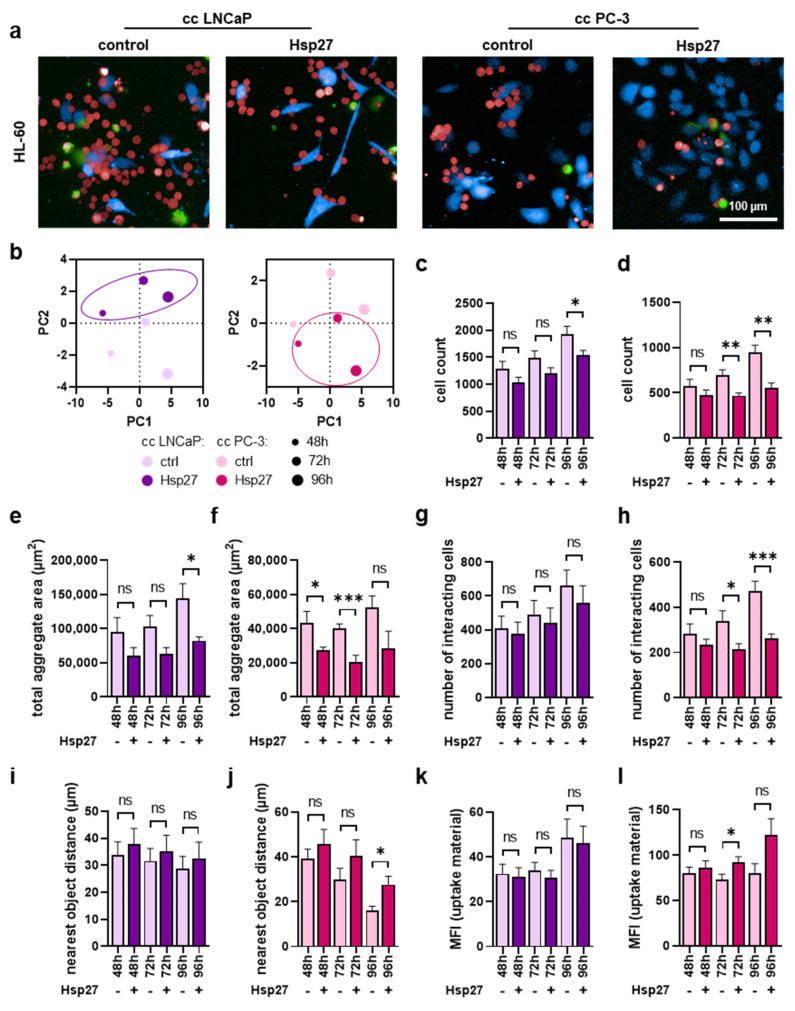
**High-content imaging of HL-60 cells co-cultured with prostate cancer cells under Hsp27 treatment.** (**a**) representative overlay images of untreated (control) and Hsp27-treated co-cultures (cc) of CellTrace Violet-labeled prostate cancer cells (blue) and CellTrace Far Red-labeled HL-60 cells (red), with SYTOX Green (green) as dead cell marker at 96 h; (**b**) PCAs of several morphology properties quantified by algorithm-based image analysis of HL-60 cells; (**c**–**l**) algorithm-based image quantification of HL-60 total cell count (**c**,**d**), the total aggregate area in µm^2^ (**e**,**f**), number of cells interacting with the prostate cancer cells (**g**,**h**), distance to the nearest prostate cancer cell in µm (**i**,**j**), and mean fluorescence intensity (MFI) of dead prostate cancer cell material internalized by HL-60 cells (**k**,**l**). Data are presented as mean + SE. Statistical analysis was performed using unpaired, two-tailed *t*-test with *p* < 0.05 (*), *p* < 0.01 (**), and *p* < 0.001 (***). ns = not significant. Scale bar is 100 µm.

Similar imaging experiments were performed using the HL60 cells (Figure 5a). Similar to THP-1 cells, morphological properties were altered by Hsp27 treatment (Figure 5b), indicating an effect of this heat shock protein within co-cultures. The number of HL-60 cells was also significantly reduced upon Hsp27 treatment in both co-cultures (Figure 5c,d). Equivalently, a significant decrease in HL-60 aggregate area (Figure 5e,f) and the number of cells interacting with prostate cancer cells (Figure 5g,h) was observed, showing an overall more pronounced effect in the PC-3 co-cultures compared to the LN-CaP co-cultures with HL-60 cells. Again, as already seen in THP-1 cells, the mean distance between HL-60 and prostate cancer cells was modestly enlarged in LNCaP (Figure 5i) and significantly increased in co-cultures with PC-3 cells (Figure 5j). The phagocytic activity was affected moderately by the Hsp27 addition to the HL-60 cells co-cultured with prostate cancer cells (Figure 5k,l).

## 4. Discussion

In this study, we aimed to investigate the role of Hsp27 in medical gas plasma-induced oxidative stress conditions with special regard to the interactions of myeloid with prostate cancer cells. Compared to untreated cells, Hsp27 induced changes in secretion profiles, activation markers, proliferation, and the direct interaction of the myeloid cells when co-cultured with prostate cancer cells.

Hsp27 release was enhanced upon the gas plasma treatment of LNCaP and PC-3 cells. Since gas plasma treatment administers a plethora of ROS [24], oxidative stress conditions are generated in the cells. Induction of Hsp27 upon oxidative stress has been observed in prostate cancer cells before [25], and protective roles were demonstrated under stress conditions, such as radiation [26], or against other apoptotic signals, for example, in androgen ablation resistance [27]. The treatment of prostate cancer cells with Hsp27 augmented metabolic activity, as quantified using a resazurin-based assay. This supports the findings that Hsp27 is cytoprotective, by inhibiting apoptosis signaling [1]. However, it was surprising that this enhanced metabolic activity was mediated without a preceding stress stimulus. This might be explained by Hsp27 interaction with signal transducers and activators of transcription 3 (STAT3), as found in LNCaP cells [28]. The transcription factor STAT3 was also shown to promote cell viability in cancer cells [29]. By contrast, the gain of metabolic activity could not be reproduced when Hsp27 was administered to the myeloid cell lines. In addition, viability and the intracellular oxidative milieu were not affected. Regarding these results, the increased metabolic activity found in co-cultures could be mainly attributed to increased prostate cancer cells mediated by the Hsp27 treatment.

Alterations in releasing cytokines, chemokines, and growth factors can indicate immunomodulation. Hence, secretion profiles of mono- and co-cultured myeloid cells were examined, revealing differential effects of Hsp27. In THP-1 and HL-60 monocultures spiked with Hsp27, a strong increase in IL-8 secretion and partial CXCL10 secretion was detected, indicating pro-inflammatory reactions. Interestingly, CXCL10 was significantly increased in THP-1 but not in HL-60 cells stimulated with Hsp27. This could be a consequence of subtle cellular activation. In activated THP-1 cells, CXCL10 levels can increase up to 1000-fold [30], while activated HL-60 cells increase CXCL10 negligibly [31], pointing to different response pathways in these cell lines from monocytic compared to granulocytic precursors, respectively. Both IL-8 and CXLC10 act as chemoattractants. In the case of CXCL10, T cells, monocytes, and NK cells are recruited [32], while IL-8 (CXCL8) is a well-known activator of neutrophils [33]. In contrast, the affect on co-cultures was more pronounced with the addition of Hsp27, showing a consistent decrease in VEGF across all conditions; decreased CXCL1 for LNCaP co-cultures, decreased IL-6 for THP-1 co-cultures, and increased IL-6 for HL-60 co-cultures. Playing an important role in tumor angiogenesis [34], VEGF reduction might be a beneficial effect of Hsp27 regarding tumor progression [35]. It is also noteworthy that the Hsp27-induced phenotype in monocultured myeloid cells was presumably (or partially) retained in co-cultured cells, as seen with IL-6 secretion, an acute phase protein with pleiotropic functions [36]. The decrease in THP-1 and increase in HL-60 monocultures was phenocopied in the co-cultures with prostate cancer cells upon Hsp27 addition. Whether this effect is due to increased consumption, or decreased or increased release, can only be speculated. Furthermore, Hsp27 could influence the prostate cancer cell secretion, masking the myeloid cell secretion changes.

Possible differentiation-like behavior of myeloid cells upon Hsp27 addition was examined by analyzing the expression of several surface markers. For myeloid cells alone, Hsp27 did not show substantial effects. It should be mentioned that THP-1 cells, but not HL-60 cells, are capable of releasing Hsp27 on their own [37,38]. In THP-1 cells, main effects were observed upon co-culture with PC-3 cells, indicating a certain amount of differentiation. Here, the markers CD11b and CD11c are associated with myeloid cell maturation [39,40]. Also associated with activation is the early marker CD69 [41]. Furthermore, an increased expression of CD163 points to the differentiation of THP-1 cells to M2-type macrophages [42]. The reduced HLA-ABC expression, which summarizes the human major histocompatibility complex class 1 (MHC-I) [43], was modest overall and possibly linked to a differentiation-like behavior in monocytes upon stimulus [44]. In HL-60 cells, differentiation-like processes upon Hsp27 addition were less pronounced. An increase in the myeloid cell maturation-associated marker CD14 [45] was found, while other surface markers typically expressed in differentiated HL-60 cells [46] were unaffected or slightly decreased. This is also supported by the increased CD71 expression, which is typically internalized in the HL-60 differentiation process to granulocytes [47]. Morphological changes identified in the myeloid cell lines were in line with the partially enhanced differentiation markers [48,49]. In addition, the decreased proliferation, as visible from the reduced myeloid cell counts, could point towards differentiation processes. Contrarily, the formation of myeloid cell aggregates and the direct interaction between myeloid cells and prostate cancer cells seemed to be impaired, suggesting a somehow attenuating effect of Hsp27 on the inflammatory response. In macrophages, Hsp27 has been shown to mediate a balance of pro- and anti-inflammatory stimuli [50], which could explain our ambiguous findings.

Considering the Hsp27 release resulting from medical gas plasma-induced oxidative stress, a potentially beneficial effect could be triggered in the cancer cells which needs to be further investigated. Moreover, basal Hsp27 expression rates of different cancer cell entities should be considered to evaluate the potential efficiency of such treatments.

## 5. Conclusions

Hsp27 affected the interplay between myeloid and prostate cancer cells. The differing secretion of pro- and anti-inflammatory signaling molecules accompanied by enhanced differentiation markers, but reduced direct interactions, indicated a bilateral role of Hsp27. A probable mitigating effect of Hsp27 protecting prostate cancer cells from myeloid cell-mediated cytotoxicity should be evaluated in further studies.

## Figures and Tables

**Table 1:** **Antibodies used for this study**.

Target	Fluorochrome	Clone	Vendor	Cat#
CD11b	APC-Cy7	ICRF44	BD Biosciences, Heidelberg, Germany	557754
CD11c	eFluor450	3.9	Thermo Fisher Scientific, Dreieich, Germany	48-0116-42
CD14	AF700	61D3	Thermo Fisher Scientific, Dreieich, Germany	56-0149-42
CD16b	PE	CLB-gran11.5	BD Biosciences, Heidelberg, Germany	550868
CD32	PE/Cy7	FUN-2	BioLegend, Amsterdam, The Netherlands	303214
CD45RA	AF700	HI100	BD Biosciences, Heidelberg, Germany	560673
CD55	PE-Cy5.5	IA10	BD Biosciences, Heidelberg, Germany	555695
CD69	BV650	FN50	BD Biosciences, Heidelberg, Germany	563835
CD71	APC	M-A712	BD Biosciences, Heidelberg, Germany	551374
CD163	PE	GHI/61	BD Biosciences, Heidelberg, Germany	556018
HLA-ABC	APC	G46-2.6	BD Biosciences, Heidelberg, Germany	555555
P2Y2	AF488	E-3	Santa Cruz Biotechnology, Heidelberg, Germany	sc-518019

## Data Availability

Available from the corresponding author upon reasonable request.
